# Brain Derived Neurotrophic Factor Interacts with White Matter Hyperintensities to Influence Processing Speed and Hippocampal Volume in Older Adults

**DOI:** 10.3233/JAD-221178

**Published:** 2023-05-02

**Authors:** Einat K. Brenner, Alexandra J. Weigand, Lauren Edwards, Kelsey R. Thomas, Emily C. Edmonds, Mark W. Bondi, Katherine J. Bangen, for the Alzheimer’s Disease Neuroimaging Initiative

**Affiliations:** a Department of Psychiatry, University of California San Diego, La Jolla, CA, USA; b Research Service, VA San Diego Healthcare System, San Diego, CA, USA; c San Diego State University/UC San Diego Joint Doctoral Program in Clinical Psychology, San Diego, CA, USA; d Banner Alzheimer’s Institute, Tucson, AZ, USA

**Keywords:** Alzheimer’s disease, brain-derived neurotrophic factor, hippocampal volume, neuropsychology, type-2 diabetes, white matter hyperintensities

## Abstract

**Background:**

Brain-derived neurotrophic factor (BDNF) is a neurotrophin that plays an important role in regulating synaptic activity and plasticity.

**Objective:**

Given that type-2 diabetes (T2DM) increases the risk of cognitive decline, and studies have suggested lower BDNF levels may be a risk factor of diabetic neurovascular complications, we sought to investigate total white matter hyperintensities (WMH) as a moderator of the effect of BDNF on hippocampal volume and cognition.

**Methods:**

Older adults without dementia from the Alzheimer’s Disease Neuroimaging Initiative (N = 454 including 49 with T2DM and 405 without diabetes) underwent neuropsychological evaluation, magnetic resonance imaging to quantify hippocampal and WMH volumes, and blood draw to assess BDNF.

**Results:**

Adjusting for age, sex, and *APOE* ɛ4 carrier status, there was a significant interaction between total WMH and BDNF on bilateral hippocampal volume in the non-T2DM group (*t* = 2.63, *p* = 0.009). Examination of main effect models with a dichotomous high/low BNDF group revealed a significant main effect for low BDNF (*t* = –4.98, *p* < 0.001), such that as WMH increased, bilateral hippocampal volume decreased. There was also a significant interaction between total WMH and BDNF on processing speed in the non-T2DM group (*t* = 2.91, *p* = 0.004). There was a significant main effect for low BDNF (*t* = –3.55, *p* < 0.001) such that as WMH increased, processing speed decreased. The interactions were not significant in the T2DM group.

**Conclusion:**

These results further elucidate the protective role that BDNF plays on cognition, as well as the cognitive effects of WMH.

## INTRODUCTION

Brain-derived neurotrophic factor (BDNF) is a neurotrophin that plays an important role in regulating synaptic activity, neurotransmission, neuronal repair, and plasticity in the central nervous system. More specifically, BDNF has been linked to learning and memory. It helps neuronal maintenance in the entorhinal cortex [1] and plays a role in regulating long-term potentiation, a type of synaptic plasticity considered as the cellular correlate of long-term memory (LTM) formation [2, 3]. Some studies have suggested that BDNF regulation specifically, and not that of other neurotrophin factors, is associated with LTM formation [4, 5].

Alzheimer’s disease (AD), the most common form of dementia among older adults, often involves synaptic and neuronal degeneration of the hippocampus, and one of the areas where BDNF is expressed is the hippocampus nuclei. In older adults with AD, BDNF plasma and serum levels have repeatedly been shown to be significantly decreased when compared with healthy older adults [6] and those with vascular dementia [7]. This decrease in BDNF may contribute to the pathogenic process of AD through lack of trophic support. One meta-analysis found that in AD, but not mild cognitive impairment (MCI)—which is conceptualized as a transitional stage between normal cognition and dementia—BDNF levels are significantly lower, suggesting that peripheral changes are more easily detected at later stages in the disease [6]. Another found that BDNF levels were significantly positively associated with CSF Aβ_42_ levels and significantly correlated with medial temporal lobe atrophy [8]. Higher levels of BDNF were correlated with lower hippocampal pro-BDNF levels and higher hippocampal p-Tau accumulation [9]. Despite the significant amount of research on BDNF levels in AD and associations with AD pathology, BDNF has not been widely studied in individuals without dementia, particularly investigating the relationship between BDNF levels and cognition.

One important factor in studying risk for decline in older adults without dementia is white matter hyperintensities (WMH), a marker for small vessel cerebrovascular disease. Total WMH have been shown to be associated with conversion from normal cognition to MCI [10], and one study found that autosomal-dominant AD is associated with increased WMH several years before symptom onset [11]. WMH may cause cognitive decline, particularly in processing speed [12], and WMH studies on the whole suggest that WMH are may contribute to the development of dementia [13]. Although the precise mechanism of the effect of WMH on AD is unknown, regional distribution and volume may play a role [14–16]. WMH are thought to be heterogeneous and have been associated with processes including demyelination, axonal loss due to ischemia or neuronal death, microglia and endothelial activation, and cerebral amyloid angiopathy [17]. The relationship between BDNF and WMH is not well studied, although one study found that number and volume of deep white matter lesions was positively associated with BDNF levels in patients without dementia [18].

Type 2 diabetes mellitus (T2DM) is a condition that increases the risk of cognitive decline, development of dementia including AD, and cardiovascular disease, a leading cause of death in people with T2DM. T2DM is also associated with deficits in multiple domains of cognitive functioning, including memory and executive functions [19, 20]. It is linked to reduced cerebral blood flow, particularly in brain regions implicated in AD such as the medial temporal lobes [21], as well as cerebrovascular disease.

The literature examining the role of BDNF in T2DM is limited but increasing, and several studies have found that the circulating level of BDNF is reduced in individuals with T2DM alone, AD alone, and more reduced in individuals diagnosed with both [22]. Lower BDNF levels have been shown to be correlated with worse delayed memory in T2DM, and there is evidence that decreased insulin resistance is associated with increased release of BDNF [23]. Several studies also suggest that lower BDNF levels may be a risk factor of diabetic neurovascular complications (for review, see [23]).

Since T2DM increases the risk of cognitive decline and development of dementia, and several studies have suggested that lower BDNF levels may be a risk factor of diabetic neurovascular complications, we sought to investigate 1) differences in BDNF levels between those with and without T2DM, and 2) WMH volume, a marker of small vessel cerebrovascular disease, as a moderator on the association of BDNF with both cognition and hippocampal volume in older adults with and without T2DM.

## MATERIALS AND METHODS

### ADNI data set

Data used in the preparation of this article were obtained from the Alzheimer’s Disease Neuroimaging Initiative (ADNI) databasehttps://adni.loni.usc.edu The ADNI was launched in 2003 as a public-private partnership, led by Principal Investigator Michael W. Weiner, MD. The primary goal of ADNI has been to test whether serial magnetic resonance imaging (MRI), positron emission tomography (PET), other biological markers, and clinical and neuropsychological assessment can be combined to measure the progression of MCI and early AD.

### Participants

All participants included in ADNI were between the ages of 55 and 90 years, had completed at least 6 years of education, were Spanish or English speakers, had Geriatric Depression Scale scores <6 (possible score range is 0–15) [24], had modified Hachinski Ischemic Scale scores <4, and were free of any significant neurological disease, major psychiatric conditions, or systemic illness. ADNI was approved by the institutional review boards at participating institutions and written informed consent was obtained. Participants were included in this study if they were not diagnosed with clinical dementia and had BDNF data available at their baseline visit. This resulted in 454 participants. Of these, 49 met criteria for T2DM and 405 did not (see Table 1 for demographics). For an additional *post-hoc* analysis, participants were classified as cognitively unimpaired (CU) or MCI according to Jak/Bondi actuarial neuropsychological MCI criteria [25].

**Table 1:** Participant demographics

	T2DM (N = 49)	Non-T2DM (N = 405)	Between-group differences
Age	74.85±5.40	74.91±7.41	*t* = –1.53, *p* = 0.128, d = 0.12
Education	15.16±3.07	15.70±2.99	*t* = –0.97, *p* = 0.335, d = 0.08
Sex	38 M; 11 F	248 M; 157 F	χ^2^ = 12.58, *p* < 0.001, V = 0.10
Race	86% White, 10% Black, 2% Asian, 2% More than one race	94% White, 3% Black, 2% Asian	χ^2^ = 20.18, *p* = 0.003, V = 0.12
Cognitive Status	18 CU; 31 MCI	147 CU; 258 MCI	χ^2^ = 2.26, *p* = 0.133, V = 0.04
*APOE* Status	18 *APOE* ɛ4 carriers; 31 non-carriers	198 *APOE* ɛ4 carrier; 207 non-carriers	χ^2^ = 0.074, *p* = 0.785, V V = –0.007
Pulse Pressure	61.21±14.81	59.65±13.81	*t* = 1.31, *p* = 0.191, d = 0.11

Welch two-sample *t*-tests were used for evaluating differences in age, education, and pulse pressure between the T2DM and non-T2DM groups. Chi-square tests were used to evaluate differences in sex, race, cognitive status, and *APOE* ɛ4 carrier status between T2DM and non-T2DM groups. “V” indicates Cramer’s V. “d” indicates Cohen’s d.

### BDNF measurement

All plasma based BDNF data were downloaded from the ADNI website https://adni.loni.usc.edu">https://adni.loni.usc.edu/">https://adni.loni.usc.edu. Detailed methods can be found online https://adni.loni.usc.edu/methods/. Briefly, blood samples were collected during baseline visit only, in the morning after an overnight fast, centrifuged to prepare plasma, and frozen on dry ice. Samples underwent an additional freeze–thaw cycle prior to quantification of BDNF. BDNF concentration was analyzed using the multiplex immunoassay panel, which is based on Luminex’s xMAP Technology by Rules-Based Medicine (RBM, Austin, TX).

### Diabetes classification

T2DM classification was determined based on the ADNI medical history database [26] or use of glucose-lowering medications [27]. Consistent with previous work in ADNI [26], the following search terms were used to identify participants with T2DM at baseline from medical history: diabetes, diabetic, insulin, insulin-dependent diabetes mellitus, and non-insulin dependent diabetes mellitus. Individuals with type 1 diabetes were excluded.

### Neuropsychological scores

Memory recall was measured by the Rey Auditory Verbal Learning Test (RAVLT) as the number of words recalled following a 30-min delay. Recognition memory was calculated from the RAVLT by subtracting false-positive errors from the number of words correctly recognized. Processing speed was measured by time to complete Trail Making Test A. Each of these measures was converted to a z-score that was adjusted for age, education, and sex based on performance of a sample of cognitively normal ADNI participants who remained cognitively normal throughout their participation in the study (*n* = 274), consistent with previously published results [28]. Memory was chosen because of its previously discussed association with BDNF and cognitive deficits in early AD. Processing speed was examined because of its sensitivity to WMH and vascular risk.

### MR image acquisition and analysis

A description of ADNI MRI imaging data acquisition and processing is available onlinehttps://www.loni.usc.edu/. All images were acquired on 1.5 T systems with 3D T1-weighted magnetization-prepared rapid gradient echo sequences in sagittal orientation. A proton density/T2-weighted fast spin echo sequence was obtained and used for quantifying WMH. The ADNI protocol was validated across platforms and all imaging sites passed scanner validation tests [29]. Hippocampal and total intracranial volumes were derived from FreeSurfer. WMH were identified on co-registered T1, T2, and PD-weighted images using an automated method that has been previously described [30, 31]. The T1 image was stripped of nonbrain tissues and nonlinearly aligned to a minimum deformation template [32, 33]. The T2- and PD-weighted images were stripped of nonbrain tissues and warped to the space of the minimum deformation template image based on the T1 alignment and warping parameters. WMH were detected at each voxel based on image intensities of the PD, T1, and T2 images, combined with a spatial prior (the prior probability of WMH occurring at a given voxel) as well as a contextual prior (the conditional probability of WMH occurring at a given voxel based on the presence of WMH at neighboring voxels). A more detailed description of this has been previously reported [34].

### Statistical analyses

Prior to analyses, data were examined for violations of assumptions of the statistical procedures employed. Age, sex, education, and *APOE* ɛ4 status (dichotomous carrier versus noncarrier) were entered into all models as covariates, and education was added when the dependent variable was cognition. Both hippocampal volume and WMH volume were divided by total intracranial volume to account for head size. WMH volume (normalized by total intracranial volume) was log transformed to normalize their non-normal distributions. Cognitive measures were Box-cox transformed to improve normality of their distributions, and outliers were removed from BDNF and hippocampal volume variables using the interquartile range method.

We first used linear regression to examine associations between BDNF level and covariates across the entire sample (collapsing those with and without T2DM). Analyses adjusted for age, sex, *APOE* ɛ4 status, and diabetes status, but did not control for the covariate when it was the outcome variable.

Differences in BDNF level between those with and without T2DM were identified using ANCOVA. We examined the interaction between total WMH and BDNF by examining the interaction between these two variables on 1) bilateral hippocampal volume and 2) cognition (i.e., memory and processing speed), within each group (T2DM and non-T2DM). When the interaction term was significant, we examined main effects within the T2DM and non-T2DM groups. For each interaction, the relevant variables were entered into a regression analysis with corresponding dependent variable and covariates. When examining main effects, BDNF was dichotomized by median split. In a posthoc analysis, we additionally controlled for use of metformin, since it decreases glucose production by increasing the insulin sensitivity of body tissues, and one of the mechanisms described for BDNF is interfering with insulin resistance. All results remained the same when controlling for metformin.

## RESULTS

### BDNF and covariates

Across the entire sample, BDNF level was significantly associated with age, such that as age increased, BDNF decreased (*t* = –2.11, *p* = 0.035). BDNF was also significantly associated with sex (*t* = 3.54, *p* < 0.001) such that females had significantly higher BDNF levels than males (M = 0.36, SD = 0.33 versus M = 0.23, SD = 0.38; *p* < 0.001). BDNF level was not associated with *APOE* status (*t* = 0.11, *p* = 0.909).

### BDNF levels between T2DM groups

ANCOVA models adjusting for age, sex, and *APOE* ɛ4 carrier status revealed that older adults with T2DM did not show reduced BDNF levels relative to those without T2DM (M = 0.17, SD = 0.40 versus M = 0.28, SD = 0.39; F = 2.73, *p* = 0.099).

### Interaction between total WMH and BDNF on bilateral hippocampal volume

**Fig. 1:**
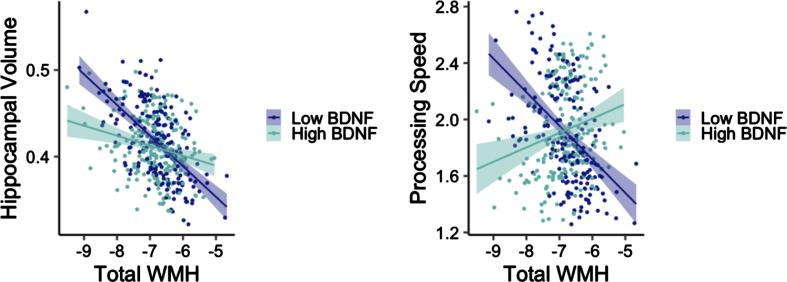
Interactions between WMH volume and BDNF on hippocampal volume (left) and processing speed (right) among older adults without diabetes. Y-axes reflect model-predicted hippocampal volume and processing speed, respectively. X-axes reflect total WMH. Hippocampal volume was normalized by intracranial volume.

Adjusting for age, sex, and *APOE* ɛ4 carrier status, there was a significant interaction between total WMH and BDNF on bilateral hippocampal volume in the non-T2DM group (*t* = 2.63, *p* = 0.009; Fig. 1). Examination of main effect models with a dichotomous high/low BNDF group revealed a significant main effect for low BDNF (*t* = –4.98, *p* < 0.001), such that as WMH increased, bilateral hippocampal volume decreased. The main effect was not significant in the high BDNF group (*t* = –0.33, *p* = 0.745). This same interaction was not significant in the T2DM group (*t* = –0.86, *p* = 0.399).

### Interaction between total WMH and BDNF on cognition

Adjusting for age, sex, APOE ɛ4 carrier status, and education, there was a significant interaction between total WMH and BDNF on processing speed in the non-T2DM group (*t* = 2.91, *p* = 0.004; Fig. 1). There was a significant main effect for low BDNF (*t* = –3.55, *p* < 0.001) such that as WMH increased, processing speed decreased. The main effect was not significant in the high BDNF group (*t* = 0.13, *p* = 0.901). This interaction was not significant in the T2DM group when examining processing speed (*t* = –1.06, *p* = 0.297), recall (*t* = –0.78, *p* = 0.440), or recognition (*t* = 0.24, *p* = 0.813). This interaction was also not significant in the non-T2DM group when examining recall (*t* = –0.24, *p* = 0.813) or recognition (*t* = –0.80, *p* = 0.427).

## DISCUSSION

Our results demonstrate that BDNF level plays a role in the associations between WMH and both hippocampal volume and cognition in those without T2DM. In our sample, older adults with T2DM did not show differences in BDNF levels relative to those without T2DM, after adjusting for demographics and dementia risk factors including age, sex, and *APOE* ɛ4 carrier status. There were significant interactions between total WMH volume and BDNF on hippocampal volume in the non-T2DM group, such that for those with low BDNF, as WMH increased, bilateral hippocampal volume decreased. There was also a significant interaction between total WMH and BDNF on processing speed in the non-T2DM group, such that for those with low BDNF, as WMH increased, processing speed decreased. These interactions were not significant in the T2DM group.

Our finding that those with T2DM had similar levels of BDNF compared to those without T2DM does not align with literature noting reduced BDNF levels in individuals with T2DM [22, 35, 36]. Importantly, one meta-analysis found that lower levels of BDNF were found in T2DM patients only when they had cognitive impairment [37]. We excluded for dementia, offering another possible explanation for these findings. Additionally, our sample had relatively low vascular risk compared to the general T2DM population because the study excluded for participants with a modified Hachinski Ischemic Scale scores >4. However, in a *post-hoc* analysis additionally controlling for cognitive status (CU versus MCI), the T2DM group had significantly lower BDNF levels (*p* = 0.047). Although everyone in the sample did not have a diagnosis of dementia, this indicates a potential effect of subtle cognitive changes on BDNF. One of the main sources of BDNF is platelets, which help regulate glucose metabolism. Low levels of BDNF have been associated with impaired glucose metabolism, and its cerebral output specifically has been shown to be negatively regulated by high plasma glucose levels [35, 38]. In people with T2DM, lower levels of BDNF were associated with obesity and diabetes complications [38]. Importantly, BDNF levels can be increased behaviorally, via exercise, which increases upregulation of BDNF as well as insulin sensitivity. Increased exercise has been linked to increases in BDNF levels, both in healthy controls and individuals with T2DM [39–41].

We also observed an interaction between total WMH and BDNF on both bilateral hippocampal volume and processing speed in individuals without T2DM. For both interactions, there were significant associations in those with low BDNF, where WMH were negatively associated with hippocampal volume and processing speed. The link between BDNF and vascular risk is not yet fully understood. However, plasma BDNF levels have been associated with risk factors for cardiovascular disease, including blood pressure, triglycerides, total cholesterol, and BMI [42]. Several studies have additionally suggested that lower BDNF levels may be a risk factor of diabetic neurovascular complications [23]. More recently, as part of the Framingham Study, high serum BDNF levels were associated with lower levels of WHM in individuals free from stroke or transient ischemic attack, and after 10-year follow-up, lower serum BDNF was associated with increased risk of incident stroke and transient ischemic attack, suggesting that BDNF levels may modify the risk of clinical and subclinical cerebrovascular disease [43]. However, studies examining the relationship between either serum or plasma BDNF and WMH in individuals with T2DM are scarce; more research is needed in this area and the precise mechanism by which BDNF affects vascular risk is unknown. It is important to note that we did not find these interactions between BDNF and WMH on cognition or hippocampal volume in the T2DM group. One possible explanation for this could be a small sample in this group (N = 49) compared to the non-T2DM group (N = 405). Another possibility is that quantifying BDNF levels using plasma may not be an optimal strategy for evaluating neurovascular complications in individuals with T2DM. Furthermore, although the volume of WMH in those with T2DM is often associated with processing speed and attention [44, 45], other research has not found these associations, including a 3-year longitudinal study [46, 47].

There are several limitations to our study worth noting. First, BDNF levels collected in ADNI are quantified in plasma, but recent literature has shown higher reliability of measurement in serum [48, 49]. Also, our sample of individuals with T2DM who had BDNF data collected was small (*n* = 49), suggesting that these findings be considered preliminary. Moreover, this sample size precluded us from conducting analyses stratified by cognitive diagnosis within participants with T2DM, however, all participants did not have dementia. All participants had modified Hachinski Ischemic Scale scores <4, indicating that they had relatively low vascular risk, likely lower than most individuals with T2DM. This may have contributed to our finding that those with and without T2DM had similar BDNF levels. Despite these limitations, our analyses add novel findings to the field.

### Conclusions

The current study examined associations between BDNF, and WMH on hippocampal volume and cognition in individuals with and without T2DM. Analyses revealed that those with T2DM had similar levels of BDNF as those without T2DM. We also observed that the association between WMH and both processing speed and bilateral hippocampal volume depends on BDNF level in individuals without T2DM. These results further elucidate the protective role that BDNF plays on cognitive decline in this population. This is suggested by the interaction between WMH and BDNF on processing speed, where, as BDNF level increases, the relationship between WMH and processing speed increases. To our knowledge, this is the first study to examine WMH and BDNF levels in non-demented individuals with and without T2DM. It contributes additional specificity, particularly in the associations between BDNF and specific cognitive domains. Future work may examine additional neurotrophins, such as insulin-like growth factor-1, which has also been shown to play a protective role in AD [50]. Future research should also investigate these relationships in individuals with AD with and without T2DM.

## Data Availability

The data supporting the findings of this study are available via request from the Alzheimer’s Disease Neuroimaging Initiative (ADNI) database https://adni.loni.usc.edu.
